# Editorial: Subjective well-being in online and mixed educational settings

**DOI:** 10.3389/fpsyg.2023.1152373

**Published:** 2023-02-24

**Authors:** Pablo Rivera-Vargas, Juan Carlos Oyanedel

**Affiliations:** ^1^Department of Teaching and Learning and Educational Organization, University of Barcelona, Barcelona, Spain; ^2^Facultad de Educación y Ciencias Sociales, Universidad Andrés Bello, Santiago, Chile

**Keywords:** COVID-19, schools, wellbeing, online learning, peer interaction, teacher-student relationships

## 1. Introduction

This Research Topic aims to bring together a set of papers that will enable the scientific community to contribute to the understanding of the changes in these factors associated with subjective wellbeing in schools. Together with this, we have sought to show how the effects of the global pandemic may not only be temporary, but a permanent change in how we understand the role of school relationships and their effects on subjective wellbeing, now actively mediated by technology.

The 24 articles that comprise it represent a significant contribution to how these changes are implemented and sustained in education systems around the world. This editorial is organized into three sections, which seek to provide a common framework for understanding Subjective Well-being in Online and Mixed Educational Settings. To this purpose, the first part presents a referential and theoretical framework of Subjective wellbeing from the social sciences in which the 24 works are inscribed. The second part describes the two thematic axes that organize the monograph and briefly presents each of the 18 articles that comprise it. Finally, we point out some considerations that we have been able to extract from the reading of the theoretical and empirical material presented in the monograph, composed of relevant voices from different geographies and traditions.

## 2. Subjective wellbeing in education

Subjective wellbeing is now a key concept in the study of human development. Its determinants and effects across the lifespan have been the subject of much research.

Over the last 20 years, interest in measuring and incorporating the “subjective wellbeing” approach has grown significantly worldwide, both in academic activity and in public policy (Calvo and Beytía, 2011; Oyanedel et al., 2015). In this regard, interest has focused on studies on adult and child populations, with little research on formal educational contexts and their school communities. At the same time, evidence on subjective wellbeing and quality of life is particularly scarce in non-Anglo-Saxon countries (Oyanedel et al., 2015), where there are no regular and comparable measurements between populations from different socio-cultural contexts (Casas et al., 2013).

The concept of quality of life is defined with respect to both objective and subjective conditions that ensure social wellbeing, with emphasis on the different stages of life development (Urzúa and Caqueo-Urízar, 2012). From the social sciences, this last element is the one that has taken on greater relevance in recent years, being defined as “subjective wellbeing.” This corresponds to the psychosocial component of quality of life, and refers to the perceptions that people have regarding their living conditions—economic, social, cultural, health, personal achievements, among others—(Seligson et al., 2003; Casas et al., 2013). Thus, it is a concept that refers to the evaluations -both positive and negative- that people have regarding their lives, from a multidimensional perspective (Lau and Bradshaw, 2010) where aspects of cognitive evaluation and emotional elaboration are integrated (Petito and Cummins, 2000) either in relation to life as a whole or to particular areas of it. Thus, with respect to quality of life, subjective wellbeing supplies the psychological devices that engage both physical and emotional stability in people's lives (Casas and Bello, 2012; Oyanedel et al., 2015), by virtue of its present but not momentary state (Veenhoven, 1994).

From the perspective of public policy, the effort to measure and monitor the subjective wellbeing of citizens has aroused greater interest because it is considered a useful tool for accessing the “fulfilled” life of a country (Ben-Arieh, 2008). This is because it complements the traditional definition of “subjective wellbeing”—measured mainly as the availability of income—, allowing development policies to be humanized by making them more inclusive (Oyanedel et al., 2015). This is precisely because “its objective is in the subjective”: considering citizens' perceptions of their lives provides fundamental information on how to improve the quality of life of this social group as beneficiaries of public policies (Ben-Arieh, 2008).

A relevant aspect in the study of subjective wellbeing is its relationship with objective indicators of economic growth and wealth in different countries. At the general population level, differences have been found according to the income of individuals, with a strong relationship when comparing countries, but decreasing when observing within countries (Oyanedel et al., 2015). Antecedents such as the above highlight the relevance of socio-economic status in relation to the study of subjective wellbeing, an area insufficiently explored from the perspective of educational communities due to the scant empirical material that existed before the COVID-19 pandemic. But this undoubtedly changed with the massive global virtualisation of educational processes that began in March 2020 (and extended in some countries to as late as mid-2021).

Research on wellbeing in educational settings has allowed us to understand the role of teachers, peer relationships, school climate and school satisfaction in subjective wellbeing. It has also helped us to assess associations of subjective wellbeing with desirable outcomes, such as higher educational achievement, and traits such as resilience, courage and self-efficacy.

Changes in educational environments as a result of the global pandemic have meant a shift in the understanding of school from a face-to-face space to a partially or fully online experience (Rivera-Vargas et al., 2021a). These changes in educational environments go beyond the mere experience of teaching and learning, changing the social relationships that underpin it (Rivera-Vargas et al., 2021b).

A relevant aspect in the study of subjective wellbeing is its relationship with objective indicators of economic growth and wealth in different countries. At the general population level, differences have been found according to the income of individuals, with a strong relationship when comparing countries, but decreasing when observing within countries (Diener and Biswas-Diener, 2002). Antecedents such as the above highlight the relevance of socio-economic status in relation to the study of subjective wellbeing, an area insufficiently explored from the perspective of educational communities due to the scant empirical material that existed before the COVID-19 pandemic. But this undoubtedly changed with the massive global virtualisation of educational processes that began in March 2020 (and extended in some countries to as late as mid-2021).

Research on wellbeing in educational settings has allowed us to understand the role of teachers, peer relationships, school climate and school satisfaction in subjective wellbeing. It has also helped us to assess associations of subjective wellbeing with desirable outcomes, such as higher educational achievement, and traits such as resilience, courage and self-efficacy.

Changes in educational environments as a result of the global pandemic have meant a shift in the understanding of school from a face-to-face space to a partially or fully online experience (Rivera-Vargas et al., 2021a; Cobo-Romani and Rivera-Vargas, 2022). These changes in educational environments go beyond the mere experience of teaching and learning, changing the social relationships that underpin it.

Schools are one of the key spaces of socialization in contemporary societies. There, children not only acquire learning and knowledge, but also social norms and develop their personalities through continuous interaction with teachers, school staff, peers and their families (Erstad et al., 2021). Most of our current knowledge about schools is based on pre-COVID-19 learning (Rivera-Vargas et al., 2021b).

The global pandemic has dramatically changed the functioning of schools from a safe space where parents could leave their children to learn to a space of potential contagion. These obvious changes are underpinned by alterations in foundational social relations, which give students a sense of purpose. These relationships are one of the key predictors of subjective wellbeing, and understanding them is key to understanding the new challenges that subjective wellbeing will now face as part of adapted educational environments.

## 3. Structure of the monograph

A total of 24 articles from the following 12 countries have been included in this monograph: Venezuela, Peru, Chile, Colombia, South Africa, China, UK, Belgium, Romania, Italy, Spain and Germany. Of these 24 articles analyzing subjective wellbeing in online and blended learning environments, 11 focused on higher education, and 13 on compulsory education (primary and secondary). It is precisely on the basis of this distinction that for this editorial we have grouped the 24 contributions into these two main blocks.

### 3.1. Higher education

In the 11 papers on higher education included in this monograph, two distinct profiles can be discerned. First, those that present the results of empirical research on the field, and second, those that present the results of the implementation of teaching innovations mediated by digital technologies.

With regard to the empirical works, we can appreciate the following:

In the article “*Emotional Wellbeing: The Impact of the COVID-19 Pandemic on Women Academics in South Africa*” written by Ronnie et al. the specific stressors manifested in South African women academics during the lockdown and their effect on emotional wellbeing are discussed. The study concludes that the work-life balance that occurred during the lockdown appeared to have a concertina effect on emotional wellbeing, as participants were pressured to manage an inordinate number of responsibilities at once.

In the article “*The Effect of Fear of the COVID-19 on Depression Among Chinese Outbound Students Studying Online in China Amid the COVID-19 Pandemic Period: The Role of Resilience and Social Support*,” authored by Chen et al. it was determined how fear of the COVID-19, correlates with depression. Along with this, the potential role of resilience and social support in the association between fear of COVID-19 and depression among Chinese students studying online in China in the midst of the COVID-19 pandemic period was explored. The results show that fear of COVID-19 was positively correlated with depression and negatively correlated with resilience and social support. Both resilience and social skills were negatively correlated with depression. Social support showed a resilience correlation.

In the article “*Emotional Intelligence and Academic Self-Efficacy in Relation to the Psychological Well-Being of University Students During COVID-19 in Venezuela*,” written by García-Álvarez et al., the predictive capacity of academic self-efficacy and emotional intelligence skills on certain dimensions of psychological wellbeing in Venezuelan university students is analyzed. The results show that emotional intelligence and academic self-efficacy are protective psychological resources for the psychological wellbeing of young university students.

In the article “*Depression, COVID-19 anxiety, subjective well-being and academic performance in university students with COVID-19 infected relatives: A Network Analysis*,” written by Ventura-León et al. examined the relationship between anxiety, depression, subjective wellbeing and academic performance in Peruvian health sciences university students with COVID-19 infected relatives. The results reveal that a depression and wellbeing node (PHQ1-SWB3) presents the highest relationship. The most central nodes belong to COVID-19 anxiety, and there are no global differences between the comparison networks; but at the local level, there are connections in the network of COVID-19 infected students that are not in the group that did not present this diagnosis.

The article “*Subjective Well-Being in Healthcare Professionals in Colombia: On the Constitution of Subjectivity and the Ethics of Care in Times of the COVID-19 Pandemic*” written by Barragán-Giraldo et al. reveal how subjective wellbeing has been generated in a group of professionals in the healthcare field in Colombia, who carried out postgraduate studies at the time of the pandemic caused by the novel SARS-CoV-2 coronavirus in a synchronous and remote learning course facilitated by employing digital technologies.

In the article “*What Matters in Online Education: Exploring the Impacts of Instructional Interactions on Learning Outcomes*” written by Li et al., the results of a research where the effects of instructional interactions on the learning outcomes of Chinese university students (i.e., academic performance and learning satisfaction) were analyzed based on the Interactive Equivalence Theory by conducting two empirical studies are presented. The results showed that task values mediated the relationship between student-content (SC) interaction and learning satisfaction. Moreover, SC may not only affect learning satisfaction directly, but also through task value and self-regulated learning respectively, or through chain mediations of both task value and self-regulated learning.

Regarding the papers on teaching innovation mediated by digital technologies, we can appreciate the following:

In the article “*Chilean University Students' Satisfaction With Online Learning During COVID-19 Pandemic: Demonstrating the Two-Layer Methodology*”, written by Montero et al., the main determinants of university students' overall satisfaction with online classes and academic performance are identified and analyzed through the domain satisfaction approach. The results show manifest student satisfaction with the support provided by the university and with learning and satisfaction with the perceived quality of online classes.

In the article “*Serious Games as a Method for Enhancing Learning Engagement: Student Perception on Online Higher Education During COVID-19*”, written by Arias-Calderón et al., the impact of the use of “serious games” as a complement to synchronous online classes to ensure the continuity of pedagogical activities in a Chilean university is analyzed. The results show that students positively valued the use of this proposed innovative pedagogical model in terms of motivation and engagement.

In the article “*Self-regulated learning and academic performance in Chilean university students in virtual modality during the pandemic: Effect of the 4Planning App*,” written by Jaramillo et al., the effect of using the 4Planning app with intracurricular focus on SRL and on the academic performance of students at a Chilean university is analyzed. The results show that students who used the app express substantial satisfaction in different pedagogical dimensions.

In the article “*Teaching Presence vs. Student Perceived Preparedness for Testing in Higher Education Online English Courses During a Global Pandemic? Challenges, Tensions, and Opportunities*,” written by Morales et al., the results of a study conducted in a Chilean university are presented in which the extent to which online teaching presence could be a mediating factor in the context of test preparation within a language course in aspects related to autonomous learning and perceived learning outcomes. However, both student and teacher voices evidenced pervasive challenges and tensions that hinder the potentially transformative benefits that online learning is expected to bring.

In the article “*E-Portfolio as an Evaluative Tool for Emergency Virtual Education: Analysis of the Case of the Andrés Bello University (Chile) during the COVID-19 Pandemic*,” written by Rodríguez et al., the results of an investigation are presented in which the perception of the students of the Phonoaudiology degree of a Chilean University on the incorporation of the E-portfolio as an evaluative tool during emergency virtual education due to the COVID-19 pandemic was analyzed. The results of the study show that there is an improvement in the methodology and teaching support, as well as in the creativity and professionalism of the students.

### 3.2. Compulsory education (primary and secondary)

In the 13 papers on compulsory education (primary and secondary) included in this monograph, two marked profiles can also be seen. Firstly, those which present a more global view of the problem, and secondly, those which present a more local view of the problem.

With regard to the works with a global view, we can appreciate the following:

In the article “*Distance Learning and School-Related Stress Among Belgians during the COVID-19 pandemic*” written by De Coninck et al., the main factors explaining increased school stress in Belgian adolescents were identified and analyzed. The results show that overcrowding, economic hardship and domestic violence are risk factors for increased stress, whereas social support and the absence of material deprivation are protective factors.

In the article “*Webcams and Social Interaction During Online Classes: Identity Work, Presentation of Self, and Well-Being*” written by Hosszu et al., a study is presented in which they analyzed how the wellbeing of teachers and students in Romania has been affected by online education through (1) the spillover effects of the sudden shift to online classes; (2) identity work at the individual and group levels; and (3) students' and teachers' presentations of self in the online environment. Results indicate that both students and teachers experienced ambivalence and various changes in wellbeing generated by the flexibility, burdens, and interruptions of homeschooling. Another aspect to note is that the identities associated with the roles of teacher and student were challenged and open to renegotiation.

The article “*Distance learning during the first confinement: impact on the family and its effect on students' engagement*” written by Chifari et al. presents research that analyzes how Distance Emergency Education (DE) impacted Italian families during the confinement caused by the COVID-19 pandemic and, in particular, to what extent the impact of DE on families, measured in terms of shared space and equipment, moderates the effect of student and family characteristics on student engagement. The main results reveal how the impact of EED on families played a significant role in predicting the level of student engagement observed by parents with respect to different predictor variables.

In the article “*Students' Experiences in Suddenly Transformed Living and Educational Environments by COVID-19*” written by Hernández-Hernández and Sancho-Gil an analysis is presented on how Spanish university students felt affected by the COVID-19 pandemic and, especially, by the irruption in this context of non-face-to-face classes and mixed teaching methods. The results show that the emotional effects have allowed them to generate positive strategies of readaptation and collaboration with other classmates.

In the article “*Well-being of School Communities in the Context of the COVID-19 Pandemic. A Qualitative Study in Chilean Schools of Low Economic Strata*”, written by López et al., presents a study whose purpose was to describe and understand the construction of school wellbeing in Chile during the pandemic, based on the notion of collective and sustainable wellbeing. The results showed that, while facing the challenges of school closures, schools made efforts to protect the wellbeing of students and teachers.

In the article “*Impact of techno-creators and techno-inhibitors on manifestations of technostress in Chilean kindergarten directors in the context of the COVID-19 pandemic and telework*”, written by Estrada-Muñoz et al., the impact of techno-creators and techno-inhibitors on the different manifestations of technostress in Chilean kindergarten directors in the context of the COVID-19 pandemic and telework is analyzed. The paper suggests that techno-creators provoke manifestations of technostress, however, techno-inhibitors did not show a significant effect in the reduction of these manifestations in the sample studied.

In the article “*Teachers' emotional exhaustion before and during the COVID-19 pandemic: Neither emotional strain nor holiday feeling*” by Bleck and Lipowsky, changes in the emotional exhaustion of active German teachers before and during the COVID-19 pandemic are analyzed. In this context, changes in the emotional exhaustion of a cohort of German professors were analyzed longitudinally, taking into account variables such as gender, age, the teaching degree studied or the amount of time devoted to distance teaching.

In the article “*Subjective Well-Being and Schools in South Africa: A Post-COVID-19 Analysis*” by Morales-Olivares et al., we present the results of a study conducted in South Africa, which analyzed subjective wellbeing in families with school-going children as a function of selected social variables such as gender and material living conditions.

In the article “*Online learning performance and engagement during the COVID-19 pandemic: Application of the dual-continua model of mental health*” by Kim et al., the results of a study in which the relationship between students' adaptation to online learning and their mental health was analyzed using the Dual-Continua Model are presented. The results revealed that two dimensions of mental health (i.e., mental wellbeing and mental disorder) were independently associated with all objective and subjective indicators of online learning.

In the article “*Psychometric properties of the Collective Efficacy Scale Short-Form in Chilean teachers*”, written by Herrera et al., the results of a study that analyzed the dimension of personal wellbeing in Chilean school teachers are presented.

Regarding the works with a local view, we can appreciate the following:

In the article “*Parental Acceptance of Educational Technology: Lessons from Around the World*” written by Osorio-Sáez et al. based on a questionnaire applied to families in 19 countries, the main factors that contribute to parents' acceptance and use of technology to support their children's learning were identified and analyzed. The results show that parents are more involved in children's learning when schools provide or suggest well-structured technological tools, and when parents are socially influenced by the opinions of other parents, teachers, children, the general public, family members, etc.

In the article “*Socioeconomic Status, Parental Involvement and Implications for Subjective Well-Being During the Global Pandemic of Covid-19*” written by Treviño et al. formal and informal parental practices of home learning during the school closure period in 19 countries around the world are analyzed. The main findings show that parental socioeconomic status is a key predictor of both formal and informal parental practices.

The article “*Psychological Well-Being in Teachers During and Post-Covid-19: Positive Psychology Interventions*” written by García-Álvarez et al. presents a systematic literature review that compiles some of the research on teacher psychological wellbeing in the context of the COVID-19 pandemic.

## 4. Final considerations

In conclusion, we believe that the contributions that form part of this Research Topic focusing on subjective wellbeing in online and blended educational settings may allow us to increase our understanding of the role of teachers, peer relationships, school climate and school satisfaction on subjective wellbeing. It can also help us assess associations of subjective wellbeing with desirable outcomes in virtual contexts, such as higher educational achievement, and traits such as resilience, courage and self-efficacy. In any case, it will be up to the educational and scientific community to judge the impact of these contributions. We will be watching.

## Author contributions

All authors listed have made a substantial, direct, and intellectual contribution to the work and approved it for publication.
